# Clinical management and impact of scarlet fever in the modern era: findings from a cross-sectional study of cases in London, 2018–2019

**DOI:** 10.1136/bmjopen-2021-057772

**Published:** 2021-12-24

**Authors:** Michael Trent Herdman, Rebecca Cordery, Basel Karo, Amrit Kaur Purba, Lipi Begum, Theresa Lamagni, Chuin Kee, Sooria Balasegaram, Shiranee Sriskandan

**Affiliations:** 1 National Infection Service, Public Health England (now UK Health Security Agency), London, UK; 2 UK Field Epidemiology Training Programme, Public Health England (now UK Health Security Agency), London, UK; 3 South London Health Protection Team, Public Health England (now UK Health Security Agency), London, UK; 4 Oak Lodge Medical Centre, Barnet, North Central London CCG, London, UK; 5 Department of Infectious Disease, Imperial College London, London, UK; 6 NIHR Health Protection Research Unit in Healthcare-associated infection and AMR, Imperial College London, London, UK; 7 MRC Centre for Molecular Bacteriology and Infection, Imperial College London, London, UK

**Keywords:** primary care, microbiology, epidemiology

## Abstract

**Objectives:**

In response to increasing incidence of scarlet fever and wider outbreaks of group A streptococcal infections in London, we aimed to characterise the epidemiology, symptoms, management and consequences of scarlet fever, and to identify factors associated with delayed diagnosis.

**Design and setting:**

Cross-sectional community-based study of children with scarlet fever notified to London’s three Health Protection Teams, 2018–2019.

**Participants:**

From 2575 directly invited notified cases plus invitations via parental networks at 410 schools/nurseries with notified outbreaks of confirmed/probable scarlet fever, we received 477 responses (19% of those directly invited), of which 412 met the case definition. Median age was 4 years (range <1 to 16), 48% were female, and 70% were of white ethnicity.

**Outcome measures:**

Preplanned measures included quantitative description of case demographics, symptoms, care-seeking, and clinical, social, and economic impact on cases and households. After survey completion, secondary analyses of factors associated with delayed diagnosis (by logistic regression) and consequences of delayed diagnosis (by Cox’s regression), and qualitative analysis of free text comments were added.

**Results:**

Rash was reported for 89% of cases, but followed onset of other symptoms for 71%, with a median 1-day delay. Pattern of onset varied with age: sore throat was more common at onset among children 5 years and older (OR3.1, 95% CI 1.9 to 5.0). At first consultation, for 28%, scarlet fever was not considered: in these cases, symptoms were frequently attributed to viral infection (60%, 64/106). Delay in diagnosis beyond first consultation occurred more frequently among children aged 5+ who presented with sore throat (OR 2.8 vs 5+without sore throat; 95% CI 1.3 to 5.8). Cases with delayed diagnosis took, on average, 1 day longer to return to baseline activities.

**Conclusions:**

Scarlet fever may be initially overlooked, especially among older children presenting with sore throat. Raising awareness among carers and practitioners may aid identification and timely treatment.

**Strengths and limitations of this study**

- We describe the clinical features and epidemiology of a disease that has seen limited observational research since the early 20th century.
- We quantify delayed diagnosis and identify factors associated with this delay and the consequences of delays for cases and their household members.
- We ascertain not only clinical consequences for participating cases, but evidence of onward transmission, time off school and time off work for carers.
- By surveying parents and guardians of notified cases, we obtain the perspective of carers on the impact of illness on the case and household (though, in consequence, we do not directly capture the perspective and rationale of clinicians).
- The survey’s low response rate highlights the risk of selection bias: participants may not fully represent the population affected by scarlet fever.

## Introduction

### Background and rationale

Improving clinical and public health management of scarlet fever depends on updating our understanding of the disease. Classical descriptions of its presentation and transmission from the early 20th century do not adequately reflect modern demographics, clinical practice or modern understanding of the pathogenesis and epidemiology of superantigen-expressing group A streptococci (GAS).^1–4^ Incidence of scarlet fever in England and Wales declined from the 1940s to the mid-2010s, but increased markedly in the 5 years leading up to the pandemic lockdown of 2020, coinciding with the emergence of a dominant, more toxigenic lineage of GAS.^5–10^

Recognising scarlet fever and commencing antibiotics and public health actions reduces the risk of sequelae and onward transmission.^10^ Scarlet fever is a notifiable infectious disease in England, usually diagnosed from symptoms and signs, with or without confirmation of GAS expressing erythrogenic toxins. The triad of fever, sore throat and rash is typical, but non-specific: this presentation may be mistaken for viral infection, or for mild pharyngitis or tonsillitis if the rash appears after other symptoms.^11^ Clinicians face a challenge in distinguishing scarlet fever and other severe manifestations of GAS infection—for which antibiotics should be commenced early—from viral upper respiratory tract infections, for which antibiotics should be avoided.

Scarlet fever has consequences not only for the infected individual, but also for their household and community. To prevent onward transmission, identified cases are excluded from school or nursery until 24 hours after starting antibiotics; this has an educational cost for the case, plus a wider economic impact on parents or guardians who provide childcare. Given the high transmissibility of GAS, household members also face a greater risk of infection. Updating our understanding of the disease includes updating our assessment of these wider consequences.

### Objectives

To inform clinical and public health practice during a surge in scarlet fever notifications in London, we surveyed the parents and guardians of cases, characterising presenting features and healthcare experience, analysing factors associated with delayed diagnosis, and identifying the health and economic impact of late recognition for cases and their households.^10 12^

## Methods

### Study design and recruitment

We sent postal invitations to an online survey (SelectSurvey V.4.0) to parents and guardians of all children under 16 with scarlet fever notified by clinicians to Public Health England (PHE) Health Protection Teams (HPTs) in 1 March 2018–31 May 2018 in London (online supplemental file 1). From 1 March 2019 to 31 May 2019, a modified version of the survey (omitting or rewording some questions, adding others) was sent to parents/guardians of notified sporadic cases and circulated to parental networks of schools or nurseries with notified outbreaks (online supplemental file 2). We did not send follow-up invitations to non-respondents. Public health management of cases and outbreaks was according to national guidelines used by HPTs.^12^

10.1136/bmjopen-2021-057772.supp1 Supplementary data

10.1136/bmjopen-2021-057772.supp2 Supplementary data

The case definition of scarlet fever matched PHE guidance definitions of confirmed or probable scarlet fever: for sporadic cases identified through statutory notification (including the index cases of suspected outbreaks), a case constituted a clinical diagnosis of scarlet fever by a health professional (with or without detection of GAS on a throat swab); in the context of an established outbreak, cases required a credible report of signs or symptoms consistent with scarlet fever with a close epidemiological link to a confirmed or probable case (with or without confirmation by a health professional).^12^

Surveys collected data on demographics, medical history, contact history, symptoms, care-seeking behaviour, diagnoses and clinical management by health professionals, impact on household caregivers, and knowledge and attitudes regarding scarlet fever on the part of the responding parent or guardian.

Participant information was provided as preamble to the survey. Participation was voluntary and anonymised. Informed consent was inferred from survey participation.

### Data analysis

Quantitative data description and analysis were performed using Stata V.14.2 and GraphPad Prism V.7.0. Study size was determined pragmatically, attempting to contact as many notified cases and schools/nurseries as possible over the course of two high-transmission seasons.

Age was collected as a continuous variable and stratified to under 5 years or 5 years and older to increase statistical power and reflect the age at which school attendance starts. Other demographic and clinical exposure variables were ascertained and analysed dichotomously. Ethnicity proportions were compared with Department for Education primary schools data for London.^13^ In assessing symptoms and signs, description was restricted to cases diagnosed by a health professional.

For analysis of variables associated with delayed diagnosis, the outcome was defined dichotomously as a case for whom scarlet fever was not considered in the differential diagnosis at the first consultation with a clinician (in the recollection of the responding parent or guardian). A logistic regression model was constructed using a stepwise, subtractive approach. Models were compared using Akaike’s information criteria and Bayesian information criteria, with likelihood ratio tests used to address ambiguous comparisons, and stratifying as required to address effect modification.

Consequences of delayed diagnosis (defined dichotomously as above) were assessed in terms of days until recovery to normal activity, days of school/nursery missed, and days of work missed by parents/guardians, constructing Cox’s proportional hazards regression models for each outcome (subject to the condition of proportionality). The model for time to recovery to normal activity was limited to data from the 2019 survey, as it was not ascertained in 2018. Missing values were addressed in regression models by introducing an additional category for unknown values of categorical variables. Cases with missing values for the outcome variables in Cox’s regression were excluded from the analysis.

### Qualitative textual analysis

Free text volunteered by respondents was coded in NVivo V.13 and Microsoft Excel using a thematic matrix for responses concerning perception of scarlet fever, and analysed to characterise experiences of the illness, accessibility of information and care, experiences of the health service, and impact on the case and their household, and identify ramifications for providers of clinical practice and health protection.

### Patient and public involvement

Parents of children who had been directly affected by severe GAS infection were involved in the design and content of the questionnaire, while parents of children with other illnesses were involved in trialling the questionnaire. Patients were not directly involved in the development of the original research question or the mode of recruitment and conduct of the study. As responses were anonymous, results cannot be directly disseminated to participants.

## Results

### Demographic characteristics of participating cases

London HPTs identified 4172 cases of confirmed or probable scarlet fever in children 0–14 years old, and 263 school/nursery outbreaks in 2018, plus 2656 cases and 147 school/nursery outbreaks in 2019. We contacted parents or guardians of 1703 cases notified March-May 2018, plus 872 cases notified March–May 2019 (along with an unknown number contacted through dissemination of invitations via parental networks in outbreak-affected schools/nurseries). Surveys were completed for 477 children (response rate 19% of those directly invited; unknown response rate from schools/nurseries), 412 of whom met the case definition (339 in 2018, and 73 in 2019). Median age was 4 years (IQR 2–6; range <1 year to 16). In 381 cases (92%), scarlet fever was diagnosed by a health professional; 31 cases (8%) had a confirmed epidemiological link to an outbreak but may not have been diagnosed by a health professional, and hence were excluded from analyses of clinical features.

Case characteristics are described in table 1. Compared to 2015 Department for Education estimates for all children in primary schools in London, responses showed a higher proportion of white participants (70% vs 42% in primary schools, p<0.001) and lower proportions of participants of Asian/Asian British (12% vs 20%, p<0.001) and black/African/Caribbean/black British ethnicity (5% vs 21%, p<0.001).^13^

**Table 1:** Demographic characteristics of participating scarlet fever cases (n=412)

Characteristics	Cases
	N (%)
Age group	
0–2 years	66 (16)
3–4 years	156 (38)
5–9 years	177 (43)
10–16 years	12 (3)
missing	1
Sex	
Female	197 (48)
Male	212 (52)
Missing/prefer not to say	3
Ethnicity	
Asian/Asian British	47 (12)
Black/African/Caribbean/Black British	21 (5)
Mixed/multiple ethnicities	48 (12)
White	287 (70)
Other	5 (1)
Missing/prefer not to say	4
School group	
Nursery/play group	167 (43)
Reception class	85 (20)
Primary school year 1	48 (12)
Primary school year 2	31 (8)
Primary school year 3	28 (7)
School beyond year 3	33 (8)
Missing/none volunteered	20
General health prior to scarlet fever	
Ever hospitalised (for any reason)	107 (26)
Follow-up in outpatient clinic	38 (9)
Chronic underlying illness*	8 (2)
Upper respiratory tract history	
≥1 episode of sore throat in preceding year	190 (49)
Previous isolation of GAS	13 (3)
Previous tonsillectomy	11 (3)

*Four report asthma; three report recurrent tonsillitis.

GAS, group A streptococci.

### Clinical characteristics

Rash was the most commonly identified symptom, reported by 89% of respondents (table 2). Fever and sore throat were more likely than rash to be noted first. Among respondents commenting on the timing of the rash relative to other symptoms, 71% (32 of 45 responding) reported the rash followed other symptoms, with a median 1-day delay (IQR 0–2.5 days; range 0–15 days). Cases with a history of recurrent sore throat were more likely to present with sore throat initially (OR 1.9, 95% CI 1.2 to 2.9, p=0.008), and substantially more likely to experience a sore throat at some point in the illness (OR 11.3, 95% CI 4.4 to 29.3, p<0.001).

**Table 2:** Reported symptoms among cases diagnosed with scarlet fever by a health professional within 4 weeks of survey completion (n=381)

Symptom	All ages	Under 5 years old	5 years and older	χ^2^ test p value
	n/total (%)	n/total (%)	n/total (%)	<5 year vs ≥5 year
**First symptom(s) noted:**				
Fever	154/338 (46)	97/180 (54)	57/158 (36)	0.001
Sore throat	132/338 (39)	48/180 (27)	84/158 (53)	<0.001
Rash	115/338 (34)	72/180 (40)	43/158 (27)	0.014
Not playing/tiredness	57/338 (17)	36/180 (20)	21/158 (13)	0.101
**Symptom ever noted:**				
Rash	336/377 (89)	187/207 (90)	149/170 (88)	0.404
Fever	327/370 (88)	184/204 (90)	143/166 (86)	0.227
Sore Throat	289/355 (81)	151/192 (79)	138/163 (85)	0.147
Tiredness	249/338 (74)	136/180 (76)	113/158 (72)	0.401
Enlarged tonsils	180/279 (65)	100/155 (65)	80/124 (65)	1
Not eating	216/338 (64)	126/180 (70)	90/158 (57)	0.013
Not playing	158/338 (47)	86/180 (48)	72/158 (46)	0.685
Headache	124/338 (37)	53/180 (29)	71/158 (45)	0.003
Pus on tonsils	101/270 (37)	54/151 (36)	47/119 (40)	0.53
Sore tongue	102/338 (30)	57/180 (32)	45/158 (28)	0.525
Stomach ache	94/338 (28)	44/180 (24)	50/158 (32)	0.141
Vomiting	77/338 (23)	48/180 (27)	29/158 (18)	0.07
Swollen tongue	52/338 (15)	28/180 (16)	24/158 (15)	0.926
Earache	50/338 (15)	21/180 (12)	29/158 (18)	0.085
Diarrhoea	38/338 (11)	24/180 (13)	14/158 (9)	0.195

Seventy per cent of respondents characterising the rash (19/27) described it as sand-papery or rough to feel, 63% (17/27) as red, 26% (7/27) as comprising small spots, 19% (5/27) as pink, 15% (4/27) as itchy, and 4% (1/27) as peeling off. Median duration of the rash was 5 days (IQR 3–8 days; range 1–14 days). 69% (18/26) reported the rash first appeared on the trunk, 19% (5/26) on the face, and 12% (4/26) on the arms and legs. Rash was identified in 89% of White cases and 90% of cases of other ethnicities (p=0.75).

The pattern of symptoms at onset varied with age. Sore throat was a more common initial symptom among cases 5 years and older (OR 3.1, 95% CI 1.9 to 5.0, p<0.001). Rash and fever were less likely at onset among cases 5 years and older (respectively OR 0.6, 95% CI 0.4 to 0.9, p=0.014; OR 0.5, 95% CI 0.3 to 0.8, p=0.001).

### Differential diagnosis and clinical management

Table 3 summarises the sources of care sought for cases. Median duration from onset of symptoms to seeing a health professional was 2 days (IQR 1–3 days; range <1–14 days). For 31% of cases, additional consultations were undertaken (with 14% requiring three or more consultations).

**Table 3:** Pathways of care for participating cases

Care pathways (among n responding)	n (%)
First source of advice (332):	
General practitioner	267 (80)
NHS Direct telephone advice	39 (12)
Walk-in centre	30 (9)
Hospital emergency department	27 (8)
Internet	26 (8)
Urgent care centre	16 (5)
Local pharmacy	14 (4)
School nurse	3 (1)
Initial differential included SF (367)	265 (72)
Repeat visit to HCW needed (380)	116 (31)
Source of second consultation (116):	
General practice	71 (61)
Emergency department	14 (12)
Urgent care centre	11 (9)
Other	5 (4)
Reason for second consultation (116):	
Child developed new symptom(s)	44 (38)
Worried that it could be scarlet fever	37 (32)
Asked to come back if not better	19 (16)
Could not take prescribed medication	6 (5)
Called back due to swab result	6 (5)
Other*	4 (3)
Hospitalised (326)	7 (2)

*Two for further investigations, two for specialist consultation.

HCW, Health Care Worker; NHS, National Health Service; SF, Scarlet Fever.

At the first consultation with a doctor, 72% of cases (268/374) had scarlet fever as the diagnosis (or part of the differential diagnosis). When the diagnosis was delayed, 60% (64/106) had their illness ascribed to a viral infection, 21% (22/106) to tonsillitis, and 13% (14/106) to pharyngitis. Throat swabs were taken from 44% of cases (148/338). Of those who knew the results of the swab, 91% (75/82) reported GAS was isolated. Antibiotic prescribing practices are described in online supplemental table S1. Ninety-three per cent of cases were prescribed an agent consistent with clinical guidelines.

10.1136/bmjopen-2021-057772.supp3 Supplementary data

### Burden and impact of disease

Eighty per cent of cases (329/402) missed school because of their illness, with a median of 3 days lost (IQR 2–4 days; range 1–14 days). 86% of cases (316/369) were treated with over-the-counter medications (such as paracetamol or ibuprofen) in addition to prescribed antibiotics. Median time from starting antibiotics to return to normal activity such as attending school or nursery was 2 days (IQR 1–4 days; range 0–8 days, asked only in 2019, with 71 respondents).

For 53% of cases (198/372), at least one carer took time off work, with a median total of 2 days taken as leave (IQR 1–3; range 0–11 days). In 23% of cases (92/398), a carer became ill themselves. In 22% (67/301), the child’s usual carers required additional help with care during the illness—provided by family members for 80%, paid professionals for 15%, and friends for 5%. In 11% of cases (37/337), other children in the household also missed school: predominantly because they were unwell themselves; less frequently because of dependence on the caregiver to transport siblings to school. In 2019, 43% (34/79) reported other unwell family members: 29 with sore throat, 10 with tonsillitis, 6 with scarlet fever, one each with cellulitis and conjunctivitis (11 households identified multiple illnesses).

### Risk factors for delayed diagnosis

In a logistic model for delayed diagnosis among 321 cases in 2018, the strongest fit was provided by variables for age (under 5 years vs 5 and older), sore throat at onset, and interaction between these variables (table 4, online supplemental table S2). No other variables affected the model fit. Among cases aged 5 years and older, those with sore throat present at symptom onset had 2.8 times the odds of a delayed diagnosis compared with those without (95% CI 1.3 to 5.8, p<0.01). Among cases aged under 5, we found no evidence of an association between sore throat and delayed diagnosis (aOR 0.6, 95% CI 0.3 to 1.5, p=0.33).

**Table 4:** Crude analysis of demographic and clinical variables associated with delayed diagnosis (diagnosis of scarlet fever not considered at first consultation with healthcare; n=374)

Variable		All cases N	Delayed diagnosis N (%)	Crude OR	95% CI	χ^2^ test p value
Age (years)	0 to 2	62	12 (19)	1	.	
	3 to 4	145	37 (26)	1.43	0.68 to 2.98	
	5 to 6	88	27 (31)	1.84	0.84 to 4.04	
	7 to 16	79	30 (38)	2.55	1.15 to 5.65	0.01*
Sex	Female	176	46 (26)	1	.	
	Male	197	60 (30)	1.24	0.79 to 1.95	0.36
Ethnicity	White	265	77 (29)	1	.	
	Mixed	44	10 (23)	0.72	0.34 to 1.53	
	Asian	41	11 (27)	0.9	0.43 to 1.88	
	Black	18	6 (33)	1.22	0.44 to 3.38	
	Other	5	1 (20)	0.61	0.07 to 5.78	0.59†
Educational setting	Nursery	156	36 (23)	1	.	
	School	200	66 (33)	1.64	1.02 to 2.65	0.04
Healthy at baseline	Yes	339	97 (29)	1		
	No	31	9 (29)	1.02	0.45 to 2.30	0.96
Past sore throat or	Yes	175	58 (33)	1		
tonsillitis	No	179	43 (24)	0.64	0.40 to 1.01	0.06
Known SF contact	Yes	125	34 (27)	1		
	No	83	30 (36)	1.51	0.83 to 2.76	0.17
Sore Throat at onset	Yes	128	42 (33)	1	.	
	No	193	44 (23)	0.6	0.37 to 1.00	0.05
Fever at onset	Yes	147	39 (27)	1	.	
	No	174	47 (27)	1.02	0.62 to 1.68	0.92
Tiredness at onset	Yes	55	68 (26)	1		
	No	266	18 (33)	0.71	0.38 to 1.32	0.28
Rash at onset	Yes	109	24 (22)	1	.	
	No	212	62 (29)	1.46	0.85 to 2.52	0.17

*χ^2^ test for trend.

†χ^2^ test for homogeneity.

SF, Scarlet Fever.

### Consequences of delayed diagnosis

Cases returned to normal activity faster when scarlet fever was considered at the first consultation (33/52; ascertained in 2019 only), with a median recovery time of 2 days from starting antibiotics when scarlet fever was considered, vs 3 when it was not, and an HR for recovery of 0.53 (95%CI 0.28 to 0.99; p=0.047; online supplemental figure S1). Cases diagnosed without delay returned to school sooner, with a median of 2 days off (246/298) and 3 days for those with delay (92/298) (HR 0.77, 95% CI 0.59 to 0.99; p=0.045). We found no difference in days of work missed by carers between the two groups, with a median of 2 days missed for both (HR 0.91, 95% CI 0.64 to 1. 29; p=0.592). Due to the phrasing of the survey, we could not distinguish between disease burden on patients who commenced antibiotics before scarlet fever was diagnosed (for another indication), and those who did so only once diagnosed.

### Qualitative synthesis

In thematic analysis of 194 free-text comments (table 5), some respondents reported reassurance that a diagnosis was made promptly by practitioners who recognised the syndrome: others were disappointed that antibiotic treatment was delayed where symptoms were attributed to viral infection. Representativeness of online resources was questioned, such as the difficulty in finding depictions of the rash on non-White skin. While some respondents noted rapid recovery and minimal impact, others recorded spread of streptococcal infections to carers and other household members, and a wider impact of the time demands and stress of providing care to unwell children.

**Table 5:** Thematic analysis of free-text comments from respondents to questionnaires, 2018–2019

Thematic analysis:	Implications for public health:	Implications for clinical practice:
**Experience of illness and care:** Perceived stigma and fear of spread in family or school. Valued leaflets shared in school outbreaks. Noted lack of representation in online materials (including lack of images of the rash on darker skin).	Need for reassurance of confidentiality. Value of rapid communication and dissemination of information during outbreaks. Importance of inclusive and diverse educational materials.	Clinical communication should take account of fears of complications, transmission and stigma. Need for clinical and parental awareness of presentation across entire population and of how the rash presents on all skin types.
**Causes of delays** Perceived link between slow communication to parents and delays in controlling outbreaks. Some observed misdiagnosis or failure to note characteristic features at early consultations; other impressed by rapid recognition and treatment by health professionals. Some experienced delays awaiting swab results.	Circulation of information through school channels can help parents engage with public health response. Communicate public health surveillance and guidance to clinicians and schools, especially during seasonal peaks and outbreaks, to aid recognition. Timely public health action may start before microbiological confirmation.	Alertness to outbreaks in households and schools can inform clinical index of suspicion. Balanced practice of sound antibiotic stewardship for childhood fevers and sore throats, with timely initiation for scarlet fever and invasive infections. Consider swabbing and issuing prescription, with clear guidance on when to start, to avoid delay.
**Consequences of delays** Worry, annoyance, and anger that late diagnosis could increase risk of complications. Carers and other household members reported secondary infections or fear of secondary infections. Wider economic and social impact of caring for children during recovery and exclusion.	Balanced messaging: treatment is important but severe complications are rare. Communicate the risk of secondary household cases: scarlet fever and other GAS infections. Calculations of disease burden should address impact on health, education and income, for entire household.	Awareness and timely identification preserve trust in practitioners. Be alert to secondary cases of scarlet fever and other GAS infections: screen for other unwell household members, and communicate risk when diagnosis is made.

GAS, group A streptococci.

## Discussion

### Summary

Undertaken at a time of increased incidence, this study provides an update on the epidemiology and presentation of scarlet fever, identifies opportunities to improve recognition, and highlights the previously unquantified burden of disease on affected households. Most cases eventually experienced fever, rash and sore throat, but older children were more likely to experience sore throat first, perhaps because younger children were less able to recognise and describe a sore throat. The sand-papery rash of scarlet fever was eventually perceived by most carers, but it tended to appear after other symptoms, a median of 1 day later. Faced with the clinical challenge of distinguishing scarlet fever from viral exanthems and other causes of fever and sore throat, an awareness of the timing and sand-papery character of scarlet fever’s rash may help practitioners make the diagnosis and commence treatment.

Practitioners should be alert to circumstances in which scarlet fever is easily overlooked. In this survey, a delay in diagnosis among older children was 2.8 times as likely when a sore throat was present at onset, with symptoms often ascribed to viral infection. Timely recognition of scarlet fever in this age group could expedite antibiotic treatment, shorten the period of infectivity, and reduce onward propagation of GAS.

Our findings highlight the interconnectedness of scarlet fever and GAS infections more widely: 43% of respondents in 2019 reported unwell family members, many with symptoms attributable to GAS (scarlet fever, pharyngitis, tonsillitis, cellulitis). Such epidemiological links are important both in assessing the full impact of the disease, and in guiding clinical and public health management. Asking about unwell contacts may lead to the diagnosis: a key consideration not only for scarlet fever, but also for other infections presenting with fever, rash and upper respiratory symptoms of major clinical and public health concern (such as measles and rubella).^14^

### Comparison with existing literature and guidance

The clinical features of scarlet fever described by respondents corroborate classical descriptions of the disease from the early 20th century, and the information contained in current UK public health and clinical guidance.^4 12 15 16^ The ability of clinicians and parents to distinguish scarlet fever from more common and less severe infections is the key to its effective treatment. Current UK clinical guidance for sore throat advises primary care physicians to give antibiotics only when a more serious condition (such as suppurative infection or sepsis) is suspected.^17^ FeverPAIN and Centor scores are validated in rapid appraisal for GAS pharyngitis, but scarlet fever falls outside their scope.^18 19^ When it appears and is recognised, the rash of scarlet fever should prompt practitioners to commence antibiotics—particularly during the spring season when incidence typically increases (from March to May in the UK).^20^ The public health importance of prompt diagnosis and treatment is underscored by the 12-fold greater risk of invasive GAS among household contacts of scarlet fever cases.^10^ Advice to avoid unnecessary antibiotics for most sore throats is valuable to antimicrobial stewardship: the caveat is that scarlet fever and other GAS infections require antibiotics to prevent complications and reduce onward spread.

In some contexts, a patient’s risk of developing autoimmune sequelae of GAS infection (acute rheumatic fever or poststreptococcal glomerulonephritis) may influence a clinician’s decision to prescribe antibiotics. For example, clinical guidance in Australia and New Zealand advises that patients at higher risk of such sequelae (such as members of some indigenous populations and children living in crowded accommodation) might warrant a lower threshold for prescribing antibiotics.^21 22^

In this study, 80% of children missed school/nursery for a median of 3 days. Time to recovery and return to school was longer when diagnosis was delayed. As the average primary school pupil misses 7.4 days a year, this increase is substantial.^23 24^ Scarlet fever affected almost 32 000 children in the UK in 2018^25^; the direct medical costs, including hospital admissions (1 in 40 case in 2014), plus the risk of secondary GAS infections, and the non-medical costs of childcare, lost education and time off of work for parents and carers, amount to a sizeable health and economic burden.^5 26^

### Strengths and limitations

By surveying notified scarlet fever cases, this study draws on the experience of patients and households accessing primary care. However, the low response rate to survey invitations highlights a risk of selection bias. Parents of cases with a more severe illness may have been more motivated to respond to the survey; in this case, failure to include mild or atypical cases could have led to overestimation of disease burden. Alternatively, if parents facing greater obstacles to care were less likely to participate, the survey may have under-represented underserved communities.

Compared with the population at risk, more cases were white than would be expected by chance. This discrepancy could represent bias in recognition or notification, given that invasive GAS infection is observed with higher incidence in ethnicities other than white.^24 27 28^ It is important that educational materials for the public and for clinicians represents the population at risk equitably: failure to depict a diverse population may prevent awareness of how the rash appears on all skin types. Respondents observed difficulty finding illustrations of the rash on non-white skin, corroborating under-representation in educational materials noted elsewhere.^29–31^ Systematic collection of data on ethnicity when conditions such as scarlet fever are notified can help identify disparities in access to care, so that they can be addressed.^32^

The timing of the survey in relation to the clinical episode presents challenges, and a risk of ascertainment bias. Surveying a parent/guardian too soon after the clinical episode would risk failing to ascertain the full burden of disease on the patient and household, if longer-term complications are not captured; surveying too late would risk recall bias, if the respondent misremembers the details of the illness and its management. A longitudinal study of cases and households affected by scarlet fever would overcome these limitations, and provide further insights into the clinical and economic impact of infection and the variables associated with adverse outcomes.

Because this survey sought the perspective of parents and carers, it did not fully capture the perspective and practice of clinicians. We report aspects of clinical management—such as differential diagnosis, isolation of GAS and choice of antibiotics—to the extent that they were known and recalled by respondents. Clinicians may also have identified subtle clinical symptoms and signs not recognised by respondents: this could account for the small number of cases with a diagnosis of scarlet fever for whom rash or fever was not reported. A parallel survey of primary care practitioners with access to medical records would corroborate these observations, reduce the likelihood of recall bias, and help identify challenges of clinical decision-making for patients with possible scarlet fever.

### Implications for practice and research

Differentiating scarlet fever from viral infections presents a clinical challenge: sore throat is common to both conditions, and the rash of scarlet fever, though characteristic, may be subtle or delayed. The challenge of keeping diagnostic algorithms and recommendations up to date is underlined further by the emergence of a new cause of acute febrile illness, namely COVID-19.^33^ When there is diagnostic uncertainty, clinical priorities include ruling out measles (for which links to known cases and vaccination history are key)^14^ and directing antibiotic therapy appropriately.

In managing outbreaks of GAS, there may be a role for molecular point-of-care tests, to guide prescribing decisions for clinically ambiguous cases where the pretest probability is high, though their use in this setting requires further evaluation.^18 34 35^ Until the sensitivity, timeliness and cost-effectiveness of diagnostic tests improve, the diagnosis of scarlet fever usually depends on clinical evaluation of symptoms and signs, in the context of current epidemiological trends, with subsequent microbiological confirmation where possible.^35^ Alertness to seasonal peaks in scarlet fever and the occurrence of local outbreaks may help set an appropriate index of suspicion.^5 36^ Increased local incidence should drive more communication between clinicians and carers about symptoms of concern (such as a sand-papery rash), so that new symptoms can be evaluated as they evolve. The need for sound antimicrobial stewardship should not preclude access to timely clinical diagnosis of scarlet fever, microbiological testing, and empirical prescribing where they are indicated.

Further research into the interplay of scarlet fever and invasive GAS at a population level will help direct diagnostic, treatment and public health strategies to reduce the impact of outbreaks. The strains of GAS that cause scarlet fever also trigger outbreaks of pharyngitis and invasive GAS infections. As such, a single case of scarlet fever may signal a larger outbreak of unrecognised GAS infections.^5 7 37^ The wider impact that controlling scarlet fever may have on the clinical and economic burden of GAS should be considered in evaluating new interventions, such as diagnostic tests and vaccines. Meanwhile, effective control of scarlet fever and GAS depends on the coordinated efforts of clinicians and public health practitioners to identify cases and outbreaks early, implement appropriate treatment and prevent onward transmission.

## Data Availability

Data are available on reasonable request. Data are available on reasonable request to the corresponding author.

## References

[1] Wong SS , Yuen K-Y . Streptococcus pyogenes and re-emergence of scarlet fever as a public health problem. Emerg Microbes Infect 2012;1:e2. 10.1038/emi.2012.9 26038416 PMC3630912

[2] Warrack JS . The differential diagnosis of scarlet fever, measles, and rubella. Br Med J 1918;2:486–8. 10.1136/bmj.2.3018.486 20769242 PMC2342078

[3] Defining the group A streptococcal toxic shock syndrome. rationale and consensus definition. The Working group on severe streptococcal infections. JAMA 1993;269:390–1. 8418347

[4] Wesselhoeft C , Weinstein L . Medical progress: scarlet fever. N Engl J Med 1945:500–37.

[5] Lamagni T , Guy R , Chand M , et al . Resurgence of scarlet fever in England, 2014-16: a population-based surveillance study. Lancet Infect Dis 2018;18:180–7. 10.1016/S1473-3099(17)30693-X 29191628

[6] Guy R , Williams C , Irvine N , et al . Increase in scarlet fever notifications in the United Kingdom, 2013/2014. Euro Surveill 2014;19:20749. 10.2807/1560-7917.es2014.19.12.20749 24698137

[7] Lynskey NN , Jauneikaite E , Li HK , et al . Emergence of dominant toxigenic M1T1 Streptococcus pyogenes clone during increased scarlet fever activity in England: a population-based molecular epidemiological study. Lancet Infect Dis 2019;19:1209–18. 10.1016/S1473-3099(19)30446-3 31519541 PMC6838661

[8] Chalker V , Jironkin A , Coelho J , et al . Genome analysis following a national increase in scarlet fever in England 2014. BMC Genomics 2017;18:224. 10.1186/s12864-017-3603-z 28283023 PMC5345146

[9] Al-Shahib A , Underwood A , Afshar B , et al . Emergence of a novel lineage containing a prophage in emm/M3 group A Streptococcus associated with upsurge in invasive disease in the UK. Microb Genom 2016;2:e000059. 10.1099/mgen.0.000059 28348855 PMC5320645

[10] Watts V , Balasegaram S , Brown CS , et al . Increased risk for invasive group A Streptococcus disease for household contacts of scarlet fever cases, England, 2011-2016. Emerg Infect Dis 2019;25:529–37. 10.3201/eid2503.181518 30602121 PMC6390732

[11] National Institute for Health and Care Excellence . Sore throat (acute): antimicrobial prescribing. NICE, 2018.

[12] Public Health England . Guidelines for the public health management of scarlet fever outbreaks in schools, nurseries and other childcare settings. Wellington House, London: PHE, 2017.

[13] Department for Education . Schools, pupils, and their characteristics, 2019.

[14] Moten M , Phillips A , Saliba V , et al . Measles: is it still a threat? Br J Gen Pract 2018;68:404–5. 10.3399/bjgp18X697961 29970395 PMC6104891

[15] Allison VD , Gunn W . The epidemiology of streptococcal infections. Proc R Soc Med 1932;25:927–44. 19988768 10.1177/003591573202500701 PMC2183778

[16] Gunn W , Griffith F . Bacteriological and clinical study of one hundred cases of scarlet fever. J Hyg 1928;28:250–66. 10.1017/s0022172400009608 PMC2167774 20474999

[17] National Institute for Health and Care Excellence . Sore throat (acute): antimicrobial prescribing (NG84). NICE guideline: NICE, 2018.

[18] McIsaac WJ , Kellner JD , Aufricht P , et al . Empirical validation of guidelines for the management of pharyngitis in children and adults. JAMA 2004;291:1587–95. 10.1001/jama.291.13.1587 15069046

[19] Little P , Hobbs FDR , Moore M , et al . Clinical score and rapid antigen detection test to guide antibiotic use for sore throats: randomised controlled trial of prism (primary care streptococcal management). BMJ 2013;347:f5806. 10.1136/bmj.f5806 24114306 PMC3805475

[20] Ebell MH , Smith MA , Barry HC , et al . The rational clinical examination. does this patient have Strep throat? JAMA 2000;284:2912–8. 10.1001/jama.284.22.2912 11147989

[21] Jaine R , Baker M , Venugopal K . Acute rheumatic fever associated with household crowding in a developed country. Pediatr Infect Dis J 2011;30:315–9. 10.1097/INF.0b013e3181fbd85b 20948456

[22] Parnaby MG , Carapetis JR . Rheumatic fever in Indigenous Australian children. J Paediatr Child Health 2010;46:527–33. 10.1111/j.1440-1754.2010.01841.x 20854325

[23] Department for Education . Pupil absence in schools in England: 2018 to 2019, 2020.

[24] Efstratiou A , Lamagni T . Epidemiology of Streptococcus pyogenes. In: Ferretti JJ , Stevens DL , Fischetti VA , eds. Streptococcus pyogenes : Basic Biology to Clinical Manifestations. Oklahoma City (OK), 2016.

[25] Public Health England . Group A streptococcal infections: first report of seasonal activity, 2018/19: health protection report: PHE, 2019.

[26] Pfoh E , Wessels MR , Goldmann D , et al . Burden and economic cost of group A streptococcal pharyngitis. Pediatrics 2008;121:229–34. 10.1542/peds.2007-0484 18245412

[27] O'Loughlin RE , Roberson A , Cieslak PR , et al . The epidemiology of invasive group A streptococcal infection and potential vaccine implications: United States, 2000-2004. Clin Infect Dis 2007;45:853–62. 10.1086/521264 17806049

[28] Hoge CW , Schwartz B , Talkington DF , et al . The changing epidemiology of invasive group A streptococcal infections and the emergence of streptococcal toxic shock-like syndrome. A retrospective population-based study. JAMA 1993;269:384–9. 8418346

[29] Lester JC , Taylor SC , Chren M-M . Under-Representation of skin of colour in dermatology images: not just an educational issue. Br J Dermatol 2019;180:1521–2. 10.1111/bjd.17608 31157429

[30] Ebede T , Papier A . Disparities in dermatology educational resources. J Am Acad Dermatol 2006;55:687–90. 10.1016/j.jaad.2005.10.068 17010750

[31] Adelekun A , Onyekaba G , Lipoff JB . Skin color in dermatology textbooks: an updated evaluation and analysis. J Am Acad Dermatol 2021;84:194–6. 10.1016/j.jaad.2020.04.084 32335181

[32] de Lusignan S , Correa A , Pathirannehelage S , et al . RCGP research and surveillance centre annual report 2014-2015: disparities in presentations to primary care. Br J Gen Pract 2017;67:e29–40. 10.3399/bjgp16X688573 27993900 PMC5198624

[33] Stokes EK , Zambrano LD , Anderson KN , et al . Coronavirus Disease 2019 Case Surveillance - United States, January 22-May 30, 2020. MMWR Morb Mortal Wkly Rep 2020;69:759–65. 10.15585/mmwr.mm6924e2 32555134 PMC7302472

[34] Shulman ST , Bisno AL , Clegg HW , et al . Clinical practice guideline for the diagnosis and management of group A streptococcal pharyngitis: 2012 update by the infectious diseases Society of America. Clin Infect Dis 2012;55:1279–82. 10.1093/cid/cis847 23091044

[35] National Institute for Health and Care Excellence . Rapid tests for group A streptococcal infections in people with a sore throat, 2019.

[36] Public Health England . Scarlet fever: symptoms, diagnosis and treatment, 2019.

[37] Brouwer S , Lacey JA , You Y , et al . Scarlet fever changes its spots. Lancet Infect Dis 2019;19:1154–5. 10.1016/S1473-3099(19)30494-3 31519542

