# Supplementary material for: Clinical management and impact of scarlet fever in the modern era: findings from a cross-sectional study of cases in London, 2018–2019
Source: BMJ Open. 2021 Dec 24;11(12):e057772. doi: 10.1136/bmjopen-2021-057772 (PMC9066343; doi:10.1136/bmjopen-2021-057772)

ONLINE SUPPLEMENTARY MATERIALS

Supplementary File 1: 2018 Online Questionnaire

Supplementary File 2: 2019 Online Questionnaire

Supplementary File 3: STROBE Checklist for Cross-Sectional Analysis

Supplementary Table S1: Antibiotic prescribing patterns described by respondents (n=339)

Antibiotic Treatment Characteristics	n	(%)
**Antibiotic Prescribed (n=311)**		
Penicillin V[1]	235	(76%)
Amoxicillin[2]	44	(14%)
Azithromycin[3]	15	(5%)
Erythromycin	14	(5%)
Other agent	6	(2%)
**Recommended Start (n=319)**		
Immediate (once prescribed)	303	(95%)
In the event of worsening symptoms	16	(5%)
**Prescribed Recommended Duration (n=288)**		
Yes	238	(83%)
No	50	(17%)
**Took Full Prescribed Course (n=339)**		
Yes	294	(87%)
No	45	(13%)
**Reason for Stopping Early (n=39)**		
Clinical improvement	16	(41%)
Doses excessive	7	(18%)
Unpleasant taste	6	(15%)
Other reasons	10	(26%)

[1]As recommended first line in NICE guidance; [2]As recommended if unable to swallow tablets; [3]As recommended if penicillin-allergic.

Supplementary Table S2: Stratified model for associations with delayed diagnosis of scarlet fever among cases in 2018 (n=321).

Stratified exposure variable	% with delayed diagnosis (n/total)	Stratum-specific adjusted OR[1]	(95% CI)	Wald test P-value	Likelihood ratio test for interaction P-value
5 years and older					
No sore throat at onset	21% (14/68)	1.00	.		
**Sore throat at onset**	**42% (34/81)**	**2.79**	**(1.34-5.82)**	**0.006**	**0.009**
Under 5 years old					
No Sore throat at onset	31% (11/35)	1.00	.		
**Sore throat at onset**	**17% (8/47)**	**0.65**	**(0.27-1.55)**	**0.328**	

Supplementary Figure S1. Recovery of scarlet fever cases among those in whom diagnosis was delayed or not delayed beyond first consultation with a health professional. Days elapsed from onset of symptoms to clinical recovery (n=52, Panel A), return to school (n=298, Panel B), and return to work for carers (n=161 Panel C).

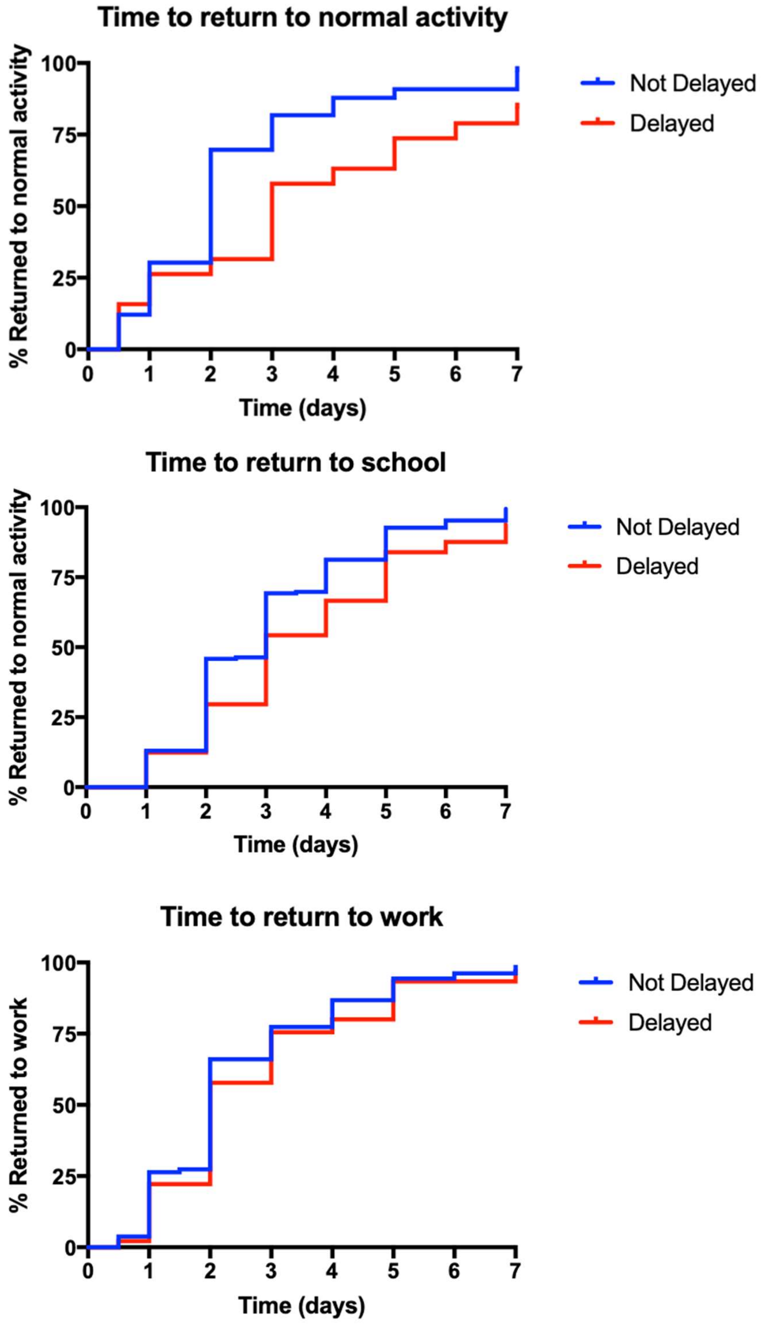

Supplement: Supplementary data [file bmjopen-2021-057772supp003.pdf]
